# The HYAL1 paradox in cancer: From complex tumor biology to novel therapeutic strategies

**DOI:** 10.1016/j.tranon.2025.102649

**Published:** 2025-12-19

**Authors:** Yun Jin, Tongyu Wang, Junyan Ma, Jiao Quan, Ning Zhou

**Affiliations:** First People's Hospital of Lanzhou City, China

**Keywords:** HYAL1, Hyaluronic acid metabolism, Tumor microenvironment, Targeted therapy, Tumor heterogeneity

## Abstract

Hyaluronidase 1 (HYAL1), a key enzyme in hyaluronic acid (HA) metabolism, exhibits a perplexing paradoxical character in tumor biology. This review systematically delineates the dual roles of HYAL1 in cancer: on one hand, by degrading HA to generate low-molecular-weight fragments with pro-angiogenic and immunomodulatory activities, HYAL1 promotes tumor progression and metastasis in various malignancies, including prostate cancer, esophageal carcinoma, and osteosarcoma; on the other hand, it demonstrates tumor-suppressive properties in specific contexts such as colorectal cancer models. This functional contradiction underscores the context-dependent nature of HYAL1, whose activity and effects are profoundly influenced by tumor type, microenvironmental features, and epigenetic regulation.

Mechanistically, HYAL1 functions not only through the classical HA-CD44 signaling axis but also by regulating the MMPs/TIMPs balance, integrin activation, and cytoskeletal reorganization. Recent studies reveal that HYAL1 expression is directly controlled by epigenetic regulators like BRD2, while its activity can be monitored in real-time using novel bioluminescent probes, providing powerful tools for investigating its dynamic functions. Notably, HYAL1-based therapeutic strategies have shown considerable promise, particularly in oncolytic virotherapy, where HYAL1-expressing recombinant viruses significantly enhance the penetration and efficacy of chemotherapeutic agents and immune cells within tumors.

Key challenges persist, including HA metabolic complexity, functional redundancy among hyaluronidases, and HA fragment instability. Future research must decipher HYAL1′s context-specific roles within tumor heterogeneity, elucidate its epigenetic regulation, and develop targeted strategies. A deeper understanding of this HYAL1 paradox is essential to leverage its potential for precision cancer therapies targeting HA metabolism.

## Highlights

• **Dual-Functionality of HYAL1**: HYAL1 exhibits paradoxical roles in cancer, acting as both a tumor promoter (e.g., in prostate, esophageal, and osteosarcoma) and a tumor suppressor (e.g., in colorectal cancer), highlighting its context-dependent nature.
• **Mechanistic Complexity**: HYAL1 influences tumor progression through multiple pathways, including HA degradation, endocytosis enhancement, MMP/TIMP balance regulation, integrin activation, and cytoskeletal reorganization.
• **Epigenetic Regulation**: HYAL1 expression is directly regulated by epigenetic factors such as BRD2, revealing a novel layer of transcriptional control in cancers like pancreatic ductal adenocarcinoma.
• **Therapeutic Potential**: HYAL1-expressing oncolytic viruses demonstrate significant promise in remodeling the tumor microenvironment, enhancing drug penetration, and improving the efficacy of chemotherapy and immunotherapy.
• **Biomarker and Diagnostic Value**: HYAL1 serves as a prognostic biomarker in multiple cancers (e.g., bladder, oral, and colorectal cancer) and is detectable in serum and urine, supporting its clinical utility.

## Graphical abstract

**Figure:**
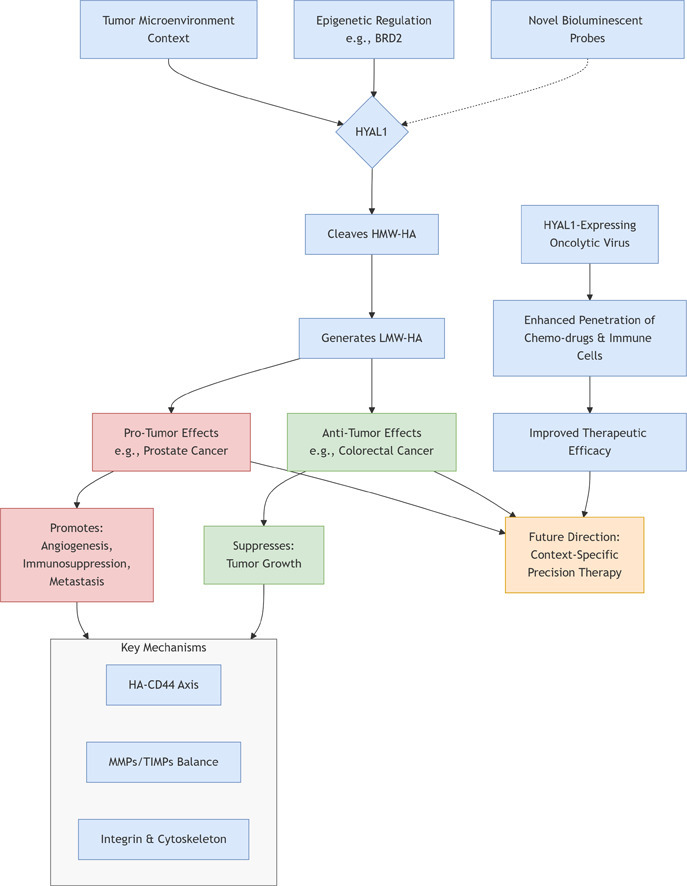

## Introduction

Hyaluronic acid (HA) is a high-molecular-weight, unbranched linear glycosaminoglycan (GAG) and a major component of the extracellular matrix. Its structure consists of repeating disaccharide units of N-acetylglucosamine and d-glucuronic acid, linked by alternating β1,3 and β1,4 glycosidic bonds [1]. Unlike other GAGs, HA is characterized by its large molecular size, absence of sulfation or epimerization modifications, and is synthesized independently of a protein via membrane-bound hyaluronan synthases, which directly release the polymer into the extracellular space [1]. HA is ubiquitously distributed throughout human tissues, though its concentration and molecular size vary widely among tissue types. Due to its exceptionally high metabolic turnover rate *in vivo*, dysregulation of HA metabolism can lead to severe pathological conditions, such as mucopolysaccharidosis type IX (MPS IX) resulting from hyaluronidase 1 (HYAL1) deficiency [2,3].

The cloning and sequencing of hyaluronidase from bee venom laid the foundation for the homology-based identification of hyaluronidases in other species [1]. These enzymes (EC 3.2.1.35), classified under glycoside hydrolase family 56, function as endo-β-N-acetylhexosaminidases that cleave the β1–4 linkage in HA through a substrate-assisted mechanism and also exhibit limited activity toward chondroitin sulfate [4]. In humans, several enzymes involved in HA degradation have been identified, including six bee venom hyaluronidase homologs (HYAL1–HYAL4, HYALP6, and PH20) and two structurally distinct G8 domain-containing proteins (CEMIP/KIAA1199 and CEMIP2/TMEM2) [1]. Among these, PH20 (SPAM1) was the first mammalian hyaluronidase identified based on its homology to the bee venom enzyme, while HYAL1 was the first somatic hyaluronidase purified from human plasma, followed by the discovery of its paralogs [5,6]. Of these enzymes, HYAL1, HYAL2, and PH20 exhibit confirmed HA-degrading activity; HYAL3 appears to lack catalytic function; and HYAL4 has been reclassified as a chondroitinase without HA-hydrolyzing capability [7]. Notably, HYAL1 is the only hyaluronidase with detectable activity in serum [1].

Cancer cells preferentially utilize anaerobic glycolysis and produce lactate even under oxygen-sufficient conditions—a phenomenon known as the Warburg effect. Studies have shown that lactate, at physiological concentrations (0–10 mM), dose-dependently stimulates HA production in fibroblasts and upregulates the expression of CD44, a transmembrane glycoprotein that serves as the primary HA receptor [8]. The accumulation of HA in the peritumoral stroma establishes a microenvironment conducive to cancer cell proliferation and migration [8]. Given that HA levels often correlate with malignancy, hyaluronidases have attracted interest as potential therapeutic targets. In Europe and the United States, hyaluronidase has been incorporated into certain anticancer regimens to reduce intratumoral pressure, enhance drug penetration, and increase chemosensitivity [9]. Recent evidence also suggests that some hyaluronidases may possess intrinsic antitumor activity. For instance, HYAL1 overexpression was shown to suppress tumorigenicity in a colon cancer model [10]. However, HA plays a dual role in cancer progression: while HA levels within the tumor cells and stroma often rise with advancing malignancy, the expression of hyaluronidases varies across cancer types—in some cases elevated relative to normal tissue, in others reduced.

As the only known serum-active hyaluronidase, HYAL1 represents a promising target for cancer therapy. Nevertheless, its precise mechanistic role in tumor initiation and progression remains incompletely understood. This review aims to systematically summarize recent advances in the understanding of HYAL1 in HA metabolism and in various cancer types, and to discuss future research directions and therapeutic prospects.

## HYAL1 structure and its role in HA metabolism

The discovery of HYAL1 dates back to 1967, when researchers first isolated an acid-active serum hyaluronidase from the neutral-active testicular hyaluronidase (PH20) [11]. This enzyme was later purified from human serum and designated HYAL1. At the genomic level, HYAL1 is located on human chromosome 3p21.3, along with HYAL2 and HYAL3, initially identified with an interval of approximately 630 kbp [12]. It is noteworthy that the 3p21.3 region is considered a potential tumor suppressor gene (TSG) locus, and HYAL1 shares sequence identity with the previously reported LuCa-1 (lung cancer 1), which frequently exhibits loss of heterozygosity or homozygous deletion in most lung cancers [12]. However, subsequent studies confirmed that Hyals are not the key or sole tumor suppressor products at this locus [[12], [13], [14]].

HYAL1 exists in multiple mRNA and protein isoforms, which may compete in the net expression of enzymatic activity [8]. Using primers including the 5′-untranslated region, RT-PCR can identify two major transcripts: the predominant mRNA contains a retained intron with multiple termination codons that prevent translation, while the second, spliced form produces the functional HYAL1 protein [15]. These two isoforms are present in most tissues but exhibit markedly different relative ratios, suggesting that HYAL1 regulation in malignancy may occur not only at the DNA level but also through RNA splicing.

At the protein level, HYAL1 cDNA encodes a 435-amino acid polypeptide. Native gel electrophoresis shows that its molecular weight is approximately 57 kDa in serum and 45 kDa in urine [11,16]. HYAL1-deficient individuals completely lack serum hyaluronidase activity [16]. The protein consists mainly of a signal peptide, a catalytic domain, an epidermal growth factor (EGF)-like domain, and three N-glycosylation sites. Its crystal structure adopts a distorted (β/α)_8_-barrel fold [17]. With an optimal pH around 4.0 and widespread tissue distribution, HYAL1 is suggested to participate primarily in the lysosomal degradation of HA [7]. Strong supporting evidence comes from the observed lysosomal accumulation of HA in tissue cells and, to a lesser extent, fibroblasts of patients with MPS IX (caused by HYAL1 deficiency) [18]. Further studies indicate that HYAL1 is secreted as a precursor, internalized via the mannose receptor pathway, and transported to lysosomes for maturation [19,20]. Additionally, HYAL1 uptake has been shown to be important for HA internalization [21].

HA undergoes extremely rapid turnover in the human body. While partial degradation occurs at the site of synthesis, the majority takes place in local lymph nodes, with the remainder entering the bloodstream and being cleared by blood-filtering organs [22]. At the cellular level, HYAL1 and HYAL2 are the two key enzymes that synergistic degrade high-molecular-weight HA into smaller fragments [1]. A previously proposed cellular catabolic model suggests that cell surface HYAL2 initially cleaves extracellular high-molecular-weight HA into intermediate fragments of approximately 50–100 sugars [23]. These fragments are then internalized into early endosomes, forming lysosomes, and further degraded by the acid-active HYAL1 into primarily tetrasaccharides [23]. However, the mechanisms controlling the accumulation of specific-sized pieces remain unclear.As research in different tissues progresses, the limitations of this model have become apparent—it fails to universally explain the unique functions of HYAL1 in HA metabolism across various tissues. For example, recent studies have shown that cell-free conditioned medium from normal human epidermal keratinocytes (NHEKs) degrades high-molecular-weight HA under weakly acidic conditions, and this activity depends on HYAL1 rather than HYAL2 [24]. Thus, the precise role of HYAL1 in HA metabolism under physiological and pathological conditions remains incompletely understood, leaving significant theoretical and experimental gaps.

In malignant tissues, HA deposition and turnover are more active and rapid. The proportion of low-molecular-weight HA fragments is often higher in tumors and cancer patients than in normal tissues. These hyaluronidase-derived HA fragments exhibit diverse biological activities. For instance, HA fragments in the 30–50 sugar range produced by aggressive bladder cancer cells are highly mitogenic to endothelial cells and strongly angiogenic, potentially supporting an invasive phenotype [8]. Similarly sized fragments can also activate tumor cell integrins, enhancing binding to intercellular adhesion molecule-1 (ICAM-1) [25]. In contrast, very small HA oligosaccharides (e.g., 6–7 sugars) have been shown to inhibit anchorage-independent growth in various tumor cells [26,27]. In summary, through its involvement in HA internalization, degradation, and regulation of fragment size, HYAL1 influences the progression of malignant tumors.

## The role of HYAL1 in osteosarcoma

Osteosarcoma, a malignant bone tumor prevalent in children and adolescents, has limited clinical treatment options owing to the absence of specific targeted therapies [28]. Osteosarcoma frequently metastasizes to the lungs, leading to a poor prognosis with a five-year survival rate of only approximately 20 % after the diagnosis of metastatic disease [29].

Recent research implicates HYAL1 in the metastatic progression of this malignancy. A study utilizing tumor spheroids to model cancer stem cells demonstrated that elevated HYAL1 expression is associated with increased metastatic potential *in vivo* [30]. Furthermore, the study identified that WNT5B can promote osteosarcoma metastasis to the lungs and liver by upregulating HYAL1, concurrently leading to the degradation of its substrate, hyaluronic acid [30]. These findings suggest that HYAL1 may play a critical role in the molecular pathways of osteosarcoma metastasis, highlighting its potential as a therapeutic target for novel strategies against metastatic spread.

## The function and mechanism of HYAL1 in prostate cancer

Clinical studies have demonstrated that elevated HYAL1 expression is significantly associated with invasive prostate cancer and serves as an independent predictor of biochemical recurrence following radical prostatectomy [31]. To investigate the underlying mechanisms, an orthotopic mouse model of prostate cancer was employed. In this model, overexpression of HYAL1—either alone or in combination with hyaluronan synthase isoforms HAS2 or HAS3—was found to accelerate HA turnover, thereby increasing the frequency of tumor development and metastasis [21,31]. Further analysis revealed that this effect was attributable, in part, to enhanced tumor cell motility and accelerated cell cycle progression [21,31]. Consistent with these *in vivo* findings, human prostate cancer cells stably overexpressing HYAL1 exhibited significantly increased migratory and proliferative capacities *in vitro* [21].

At the molecular level, studies have revealed that HYAL1-overexpressing cells exhibit significant alterations in surface marker expression, including upregulation of β1-integrin and N-cadherin, along with enhanced adhesion to extracellular matrix components such as fibronectin and collagen [32,33]. Notably, the pro-proliferative and pro-migratory effects of HYAL1 depend critically on its catalytic activity and are potentially modulated by its substrate-binding affinity and ambient substrate concentrations. Furthermore, the vesicular localization of HYAL1 within prostate cancer cells has also been shown to require its enzymatic function [21].

Further mechanistic studies have revealed a novel function of HYAL1 in regulating cellular endocytosis. Overexpression of HYAL1 significantly enhances the internalization capacity of cells, affecting not only its physiological substrate HA but also non-substrate molecules such as transferrin [21]. Importantly, this broad enhancement in endocytic activity is strictly dependent on the catalytic activity of HYAL1 [21]. Researchers have further delineated the intracellular trafficking route of HYAL1 in prostate cancer cells: the enzyme is initially secreted into the extracellular space via the classical ER-Golgi pathway, then re-internalized through endocytosis and incorporated into the vesicular sorting system [21]. While a small fraction is recycled back to the plasma membrane via the Rab11-mediated slow recycling pathway, the majority of HYAL1 ultimately resides in Rab5-positive early endosomes or Rab7-positive late endosomes/lysosomes [21].

From a functional perspective, catalytically active HYAL1 enhances the endocytic efficiency of tumor cells, enabling more effective acquisition of external resources. For instance, the increased internalization of HA facilitates cellular uptake of sugars, which may subsequently influence gene expression and drive metabolic reprogramming. Following internalization in complex with HYAL1, HA is processed through two primary pathways: one portion is completely degraded into tetrasaccharides within lysosomes, while another may remain bound to HYAL1 during vesicular sorting [21]. This binding delays vesicle recycling and leads to subsequent resecretion of the complexes. These partially processed HA fragments exhibit enhanced affinity for cell surface HA receptors. Since low-molecular-weight HA fragments have been demonstrated to promote proliferation, migration, and angiogenesis, HYAL1-mediated excessive HA uptake and partial degradation may ultimately lead to oligosaccharide accumulation, thereby reinforcing a pro-tumorigenic phenotype [21].

In summary, recent studies have established that the catalytic activity of HYAL1 is essential for its ability to promote cell proliferation and migration. The underlying mechanism involves ligand binding-dependent regulation of protein localization and trafficking. In aggressive prostate tumor cells, overexpression of wild-type HYAL1 enhances the internalization and accumulation of HA fragments while augmenting global endocytic rates. These changes subsequently alter the availability and activation of cell surface receptors, ultimately leading to metabolic reprogramming and accelerated tumor progression.

## HYAL1 in colorectal cancer (CRC)

Colorectal cancer (CRC) ranks among the most common and lethal malignancies worldwide [34]. Approximately 20 % of patients present with metastatic disease at initial diagnosis, and up to 50 % of those with localized lesions will eventually develop metastases [35]. This clinical reality underscores the urgent need to identify reliable prognostic biomarkers and therapeutic targets.

A recent bioinformatic study integrating data from TCGA and GEO databases focused on colorectal mucinous adenocarcinoma (MAC). The analysis identified three distinct molecular subtypes and established a risk-score signature based on six genes (CALB1, MMP1, HOXC6, ZIC2, SFTA2, and HYAL1). This model revealed that high expression of CALB1, HOXC6, ZIC2, and SFTA2 correlated with poor patient prognosis, whereas elevated levels of MMP1 and HYAL1 were associated with favorable outcomes [36]. These findings indicate that these six genes collectively form a molecular signature strongly linked to CRC prognosis. Further validation demonstrated that patients with low risk-scores consistently exhibited better survival compared to high-risk patients, regardless of gender, age, tumor location, or TNM stage [36].

Of particular interest is the potential tumor-suppressive role of HYAL1. Evidence suggests that HYAL1 may inhibit CRC metastasis by modulating the balance between matrix metalloproteinases (MMPs) and their tissue inhibitors (TIMPs), and by reorganizing the distribution of F-actin in the cytoskeleton [37,38]. However, the precise biological functions and underlying mechanisms of HYAL1 and other signature genes in colorectal MAC remain largely unexplored experimentally, representing an important direction for future fundamental research.

## The role of HYAL1 in esophageal cancer

Esophageal cancer (EC) ranks as the eighth most common malignancy globally [39]. It is histologically classified into two main subtypes: esophageal squamous cell carcinoma (ESCC) and esophageal adenocarcinoma (EAC), with ESCC accounting for the vast majority of EC cases worldwide [40].

Recent studies have demonstrated that HYAL1 is highly expressed in ESCC tissues, cell lines, and tumor-derived exosomes [41]. To investigate its functional mechanism, researchers established a co-culture system using Transwell chambers containing ESCC cells and macrophages. Exosomes isolated from conditioned medium of cancer cells were characterized by transmission electron microscopy and Western blot, and subsequently used to treat macrophages. Experimental results showed that exosomes derived from HYAL1-overexpressing ESCC cells suppressed M1 macrophage polarization while promoting M2 polarization, thereby enhancing ESCC cell viability, invasion, and migration [41]. Conversely, HYAL1 knockdown in ESCC cells produced opposite effects on macrophage polarization and cancer cell malignancy.

Mechanistically, HYAL1 was found to interact with Aurora kinase B (AURKB), leading to activation of the PI3K/AKT signaling pathway in macrophages [41]. This interaction may represent a key molecular mechanism through which HYAL1 modulates the tumor microenvironment.

## The complex role of HYAL1 in pancreatic ductal adenocarcinoma (PDAC)

Pancreatic ductal adenocarcinoma (PDAC) ranks among the most lethal malignancies and is projected to become the second leading cause of cancer-related deaths by 2030 [42]. A hallmark of PDAC is its highly desmoplastic tumor microenvironment, where stromal components may constitute up to 90 % of the tumor mass [43,44]. This collagen- and hyaluronic acid (HA)-rich stroma actively drives tumor progression and confers therapeutic resistance [43,44]. Clinical observations consistently demonstrate that abnormal HA accumulation correlates significantly with enhanced invasiveness and treatment failure [45].

The regulation of HYAL1 expression in PDAC exhibits considerable complexity. A hypoxia-focused study revealed that while hypoxic conditions upregulate KIAA1199 expression, they simultaneously suppress HYAL1 [46]. More significantly, a systematic investigation utilizing siRNA and small-molecule inhibitors demonstrated that HYAL1 expression is markedly elevated in human PDAC tumors compared to normal pancreatic tissues, whereas HYAL2 and HYAL3 show no substantial differences [47]. This cell-type-specific expression pattern was further confirmed across various cell lines, with HYAL1 mRNA being specifically upregulated only in PDAC-derived lines [47].

Mechanistic exploration revealed that BRD2, a BET family protein, serves as a key transcriptional regulator of HYAL1. siRNA-mediated knockdown experiments demonstrated that BRD2 depletion most effectively reduced HYAL1 mRNA and protein levels across multiple cell lines. Chromatin immunoprecipitation assays confirmed that BRD2 directly binds to the HYAL1 promoter, indicating that HYAL1 transcription is subject to precise epigenetic regulation, including promoter methylation and histone acetylation [47]. Functionally, HYAL1 knockdown not only suppressed PDAC cell proliferation but also significantly induced caspase-3 and PARP cleavage, suggesting that HYAL1 deficiency promotes tumor cell apoptosis [47]. These findings systematically elucidate the pro-tumorigenic role of HYAL1 in PDAC and identify BRD2 as its crucial upstream regulator, providing a theoretical foundation for targeting HYAL1 in therapeutic strategies.

Integrating current evidence, we note a paradoxical phenomenon: although HYAL1 is generally overexpressed in PDAC, the characteristic hypoxic tumor microenvironment may suppress its expression. Given that PDAC features prominent stromal hyperplasia and hypoxia, the actual function of HYAL1 during tumor progression might be compromised or masked by compensatory mechanisms involving other hyaluronidases such as KIAA1199. This complexity may account for discrepancies in current assessments of HYAL1 functionality.

Furthermore, multiple preclinical studies support the tumor-promoting role of HA in PDAC progression. Analysis of primary pancreatic cancer specimens revealed the highest HA levels at the tumor-normal tissue interface, suggesting its involvement in local invasion [48]. Correspondingly, in PDAC cell lines including NOR-P1, CFPAC1, and Capan-2, reduced HA production was associated with increased HYAL1 mRNA expression, further indicating that HYAL1-mediated HA metabolism plays an important yet complex regulatory role in PDAC malignant progression [49].

## The role of HYAL1 in breast cancer and brain metastasis

Breast cancer is the second most common source of brain metastases (BM) [50]. The development of breast cancer brain metastasis (BCBM) severely compromises patients' quality of life and leads to a marked deterioration in prognosis. Studies indicate that patients with triple-negative breast cancer (TNBC) and HER2-positive subtypes face a higher risk of developing BM [51,52]. Notably, correlations exist between molecular subtypes of breast cancer and hyaluronic acid (HA) metabolism [[51], [52], [53]]. For instance, hormone receptor-positive cells exhibit lower endogenous HA levels compared to TNBC cells, suggesting a potential role for HA metabolism in breast cancer pathogenesis and progression [52,53].

Accumulating evidence indicates a positive correlation between HYAL1 levels and the risk of BM formation in breast cancer [54]. Recent research has demonstrated that HYAL1 and its catalytic product, low-molecular-weight HA (LMW-HA), play crucial roles in forming a pericellular HA coat around breast cancer cells [55]. This structure promotes tumor cell adhesion to brain endothelial cells, disrupts endothelial integrity, facilitates transendothelial migration *in vitro*, and ultimately accelerates brain metastasis *in vivo* [55]. Further mechanistic investigation revealed that this process depends on the HA receptor CD44—knockdown of CD44 effectively suppresses these pro-metastatic phenotypes [55]. These findings suggest that targeting the HYAL1–LMW-HA–CD44 signaling axis could represent a promising therapeutic strategy for breast cancer patients at high risk of brain metastasis.

Beyond this mechanism, several advances have been made in understanding HYAL1 regulation in breast cancer. One study found that salicylic acid broadly influences the transcription of HA metabolism-related molecules (including HAS, HYALs, and CD44) in MDA-MB-231 cells, reducing HYAL1 levels by approximately 40 %, indicating its potential as a modulator of HA metabolism [56]. Another study comparing mRNA levels of HA metabolic components between tumor and adjacent non-tumor tissues revealed significantly lower HYAL1 mRNA in breast cancer tissues [57]. The significance of this finding extends beyond mere expression levels and touches upon HA metabolic patterns: the concurrent decrease in HYAL1 and total HA levels in tumor tissues suggests that alterations in HYAL1 expression may not directly determine the overall HA accumulation burden in breast cancer, but rather modulate the functional state and biological activity of HA molecules.

## The diverse roles of HYAL1 in other types of cancer

Beyond the cancers previously discussed, HYAL1 exhibits functional diversity across various other malignancies. In male genitourinary cancers such as bladder cancer and aggressive tumors like laryngeal carcinoma, HYAL1 has been demonstrated to actively participate in tumor progression.

Although HYAL1 generally exhibits pro-tumorigenic properties in most cancers, its functions display significant context-dependency. For instance, in colon cancer models, HYAL1 overexpression surprisingly suppressed tumor formation, suggesting potential tumor-suppressive capabilities [58]. Notably, this inhibitory effect was observed *in vivo* but not under *in vitro* conditions, indicating that its anti-tumor activity may depend on comprehensive regulation within the tumor microenvironment. Recent investigations have further elucidated HYAL1′s complex roles across different cancers: in thyroid tumors, alterations in HYAL1 methylation status and expression levels show promise as novel biomarkers for distinguishing malignant from benign nodules; in melanoma, fibroblast-derived matrices treated with HYAL1 or low-molecular-weight HA-functionalized 3D collagen matrices concentration-dependently promoted tumor cell proliferation and invasion; whereas in ovarian cancer, HYAL1 expression was significantly downregulated in tumor tissues compared to normal controls [[59], [60], [61]].

From a translational perspective, the clinical value of HYAL1 is gradually emerging. In oral cancer, single-cell RNA sequencing analyses identified TGFBI and HYAL1—expressed by cancer-associated fibroblasts and endothelial cells—as robust prognostic biomarkers [62]. For bladder cancer, meta-analyses support the combination of HYAL1 and SURVIVIN as effective urinary diagnostic biomarkers [63]. Furthermore, regarding mechanistic interventions, natural polyphenols including Plumbagin, Pongapin, and Karanjin significantly downregulated HYAL1–4 expression in cervical cancer HeLa cells, with HYAL1 suppression being most prominent [64]. These compounds likely reduce low-molecular-weight HA production through coordinated regulation of HAS2 and HYAL1 expression, thereby attenuating HA-CD44 signaling pathway activity and ultimately inhibiting tumor growth.

## Targeting HA to improve therapy: From rationale to application

Excessive deposition of extracellular matrix components, such as hyaluronic acid (HA), in the tumor microenvironment (TME) impedes the penetration and distribution of antitumor drugs [65,66]. HA not only regulates tumor cell growth and invasion but has also been shown to compromise the efficacy of chemotherapeutic agents [65,66]. Furthermore, abnormal HA accumulation in the TME physically restricts the effective spread of viruses within tumor tissues [67]. Therefore, targeted degradation of HA to remodel the TME structure is expected to create favorable conditions for the intratumoral delivery and efficacy of various anticancer agents.

The above mechanism provides a theoretical basis for the application of HYAL1 as an HA-degrading enzyme in tumor therapy. In fact, hyaluronidase has long been incorporated into European clinical protocols as a chemosensitizer, primarily by reducing intratumoral pressure and enhancing drug penetration to improve chemotherapy outcomes. With growing insights into HYAL1′s functional mechanisms and the continuous development of novel anticancer therapies, HYAL1 has recently demonstrated breakthrough potential in oncology.

Taking oncolytic virotherapy as an example, this approach utilizes genetically engineered viruses that selectively replicate in tumor cells or enhance antitumor immune responses [68,69]. A recent study constructed a recombinant oncolytic vaccinia virus expressing HYAL1 (OVV-Hyal1) and systematically evaluated its TME-remodeling and antitumor effects in multiple mouse xenograft models. The results demonstrated that OVV-Hyal1 effectively degrades HA in the TME, thereby significantly promoting intratumoral viral spread, chemotherapeutic drug penetration, and immune cell infiltration and activation [70]. Notably, this strategy markedly enhanced the antitumor efficacy of chemotherapy, peptide-based therapy, CAR-T cell therapy, and immune checkpoint blockade, showing strong translational potential and laying a solid foundation for future clinical trials in solid tumors.

## Conclusions and future perspectives

Hyaluronic acid metabolism plays a pivotal role in tumor progression, and HYAL1, as a central component of this metabolic pathway, is of great significance for understanding the mechanisms of cancer development. Substantial evidence indicates that elevated HA levels in the extracellular matrix of various solid tumors—including carcinomas and sarcomas—are closely associated with malignant progression, and differential expression of HYAL1 is considered a key contributing factor. However, research on HA metabolism and the biological functions of HYAL1 has not yet formed a systematic theoretical framework. Current mechanistic insights remain fragmented, with studies predominantly focused on genitourinary and certain highly aggressive tumors, leaving considerable gaps in other malignancies.

Notably, HYAL1 influences tumor progression not only by regulating total HA content but also through the unique biological activities of its degradation product, low-molecular-weight HA fragments, which directly modulate tumor cell behavior. Therefore, in-depth investigation into the role of HYAL1 in cancer will not only advance our understanding of the microscopic mechanisms of tumor progression but also promote an integrated view of HA metabolism under both physiological and pathological conditions.

## Current research challenges

HYAL1 exhibits a perplexing dual role in cancer: it demonstrates pro-tumor properties in some aggressive cancers yet displays anti-tumor effects in specific therapeutic contexts. This functional paradox underscores its complex role within the tumor microenvironment. Several key challenges impede further progress:

First, the high turnover rate of HA *in vivo*, the dual regulation of its synthesis and degradation, and the functional redundancy and competition among different hyaluronidase family members collectively complicate the accurate dissection of HYAL1 function within relatively static experimental systems.

Second, the functional scope of HYAL1 extends beyond modulating HA accumulation. The low-molecular-weight HA fragments it generates, despite their significant bioactivity, are difficult to track due to poor stability and short half-lives. Moreover, these fragments may originate not only from the enzymatic cleavage of high-molecular-weight HA but also from direct synthesis by hyaluronan synthases. This multiplicity of sources further complicates mechanistic studies.

## Future research directions

Despite these challenges, emerging technologies offer powerful tools for HYAL1 research. For example, a recently developed non-covalent caged firefly luciferin-based bioluminescent probe enables dynamic visualization of endogenous HYAL1 activity in live animals and at the cellular level, with the ability to distinguish normal from cancerous cells [71]. This innovation should greatly facilitate the assessment of HYAL1 function in biological processes.

Based on the current research landscape, the following directions warrant emphasis:

1. **Deepening Mechanistic Insights:** Current studies predominantly focus on the effects of HYAL1 expression differences on ECM composition and tumor cell phenotype. There remains a pressing need to explore its upstream regulatory mechanisms and downstream signaling pathways.
2. **Accounting for Tumor Heterogeneity:** HA metabolism undergoes dynamic changes during tumor progression, and HYAL1 function is likely to vary significantly across cancer stages and molecular subtypes. There is a lack of fine-grained analysis of HYAL1 function tailored to specific cancer subtypes and disease stages.
3. **Epigenetic Regulation:** The potential influence of epigenetic modifications on HYAL1 expression and function across different cancers remains an open and promising field for exploration.

## Concluding remarks

The widespread presence of HYAL1 across multiple cancer types and its functional duality underscore its considerable potential in future cancer therapy—whether as a novel target for immunotherapy or as a chemosensitizing agent. However, translating this potential into clinical value necessitates a clear understanding of the context-dependent mechanisms of HYAL1 across different tumor types and microenvironments. Achieving this goal will require interdisciplinary collaboration and innovative technological platforms, which in turn will lay a solid foundation for developing precise HA metabolism-targeted strategies.

## CRediT authorship contribution statement

**Yun Jin:** Conceptualization, Investigation, Visualization, Writing – original draft. **Tongyu Wang:** Conceptualization, Investigation, Writing – original draft, Writing – review & editing. **Junyan Ma:** Investigation, Writing – review & editing. **Jiao Quan:** Investigation, Visualization. **Ning Zhou:** Funding acquisition, Project administration, Supervision, Writing – review & editing.

## Declaration of competing interest

The authors declare that they have no known competing financial interests or personal relationships that could have appeared to influence the work reported in this paper.

## References

[1] Stephen P., Barbara T.-R (2024). Genetic deficiencies of hyaluronan degradation. Cells.

[2] TC, L. & J R., Hyaluronan F.. FASEB Journal 6(1992).

[3] Kim L., Kevin M. (2021). Update in the mucopolysaccharidoses. Semin. Pediatr. Neurol..

[4] Lara G. (2012). Hyaluronidase 1 and β-hexosaminidase have redundant functions in hyaluronan and chondroitin sulfate degradation. J. Biol. Chem..

[5] M G., G K. (1993). Bee venom hyaluronidase is homologous to a membrane protein of mammalian sperm. Proc. Natl. Acad. Sci. u S. a.

[6] AB C. (1998). The hyaluronidase gene HYAL1 maps to chromosome 3p21.2-p21.3 in human and 9F1-F2 in mouse, a conserved candidate tumor suppressor locus. Genomics.

[7] Tu Anh N. (2024). Substitution of acidic residues near the catalytic Glu131 leads to human HYAL1 activity at neutral pH via charge-charge interactions. PLoS. One.

[8] Robert S. (2008). Hyaluronidases in cancer biology. Semin. Cancer Biol..

[9] J K., H S., W R., S W., J S. (1998). Hyaluronidase as additive to induction chemotherapy in advanced squamous cell carcinoma of the head and neck. Cancer Lett..

[10] Annica J., Mehdi R., Kristofer R., Paraskevi H. (2002). Expression of hyaluronan synthase 2 or hyaluronidase 1 differentially affect the growth rate of transplantable colon carcinoma cell tumors. Int. J. Cancer.

[11] GI F., AB C., T W., R S. (1997). Purification, cloning, and expression of human plasma hyaluronidase. Biochem. Biophys. Res. Commun..

[12] MI L., JD M. (2000). The 630-kb lung cancer homozygous deletion region on human chromosome 3p21.3: identification and evaluation of the resident candidate tumor suppressor genes. The International Lung Cancer chromosome 3p21.3 tumor suppressor gene consortium. Cancer Res..

[13] Debora A. (2007). Molecular analysis of deletions in human chromosome 3p21 and the role of resident cancer genes in disease. Brief. Funct. Genomic. Proteomic..

[14] Lin J. (2002). Expression of several genes in the human chromosome 3p21.3 homozygous deletion region by an adenovirus vector results in tumor suppressor activities in vitro and in vivo. Cancer Res..

[15] Nanna J., Scilla L., Lone N., Paul E.G. (2003). Expression and regulation patterns of hyaluronidases in small cell lung cancer and glioma lines. Oncol. Rep..

[16] AB C., GI F., T W., R S. (1997). Purification and microsequencing of hyaluronidase isozymes from human urine. FEBS Lett..

[17] Chao K., Muthukumar L., Herzberg O. (2007). Structure of human hyaluronidase-1, a hyaluronan hydrolyzing enzyme involved in tumor growth and angiogenesis. Biochemistry.

[18] Natowicz M. (1996). Clinical and biochemical manifestations of hyaluronidase deficiency. N. Engl. J. Med..

[19] Puissant E. (2014). Subcellular trafficking and activity of hyal-1 and its processed forms in murine macrophages. Traffic..

[20] Boonen M., Puissant E., Gilis F., Flamion B., Jadot M. (2014). Mouse liver lysosomes contain enzymatically active processed forms of hyal-1. Biochem. Biophys. Res. Commun..

[21] McAtee C. (2015). Hyaluronidase Hyal1 increases tumor cell proliferation and motility through accelerated vesicle trafficking. J. Biol. Chem..

[22] Fraser J., Kimpton W., Laurent T., Cahill R., Vakakis N. (1988). Uptake and degradation of hyaluronan in lymphatic tissue. Biochem. J..

[23] Stern R. (2004). Hyaluronan catabolism: a new metabolic pathway. Eur. J. Cell Biol..

[24] Abe M., Masuda M., Mizukami Y., Inoue S., Mizutani Y. (2024). Epidermal keratinocytes regulate hyaluronan metabolism via extracellularly secreted hyaluronidase 1 and hyaluronan synthase 3. J. Biol. Chem..

[25] Fujisaki T. (1999). CD44 stimulation induces integrin-mediated adhesion of colon cancer cell lines to endothelial cells by up-regulation of integrins and c-met and activation of integrins. Cancer Res..

[26] Stern R., Jedrzejas M. (2006). Hyaluronidases: their genomics, structures, and mechanisms of action. Chem. Rev..

[27] Ghatak S., Misra S., Toole B. (2002). Hyaluronan oligosaccharides inhibit anchorage-independent growth of tumor cells by suppressing the phosphoinositide 3-kinase/Akt cell survival pathway. J. Biol. Chem..

[28] Bielack S., Hecker-Nolting S., Blattmann C., Kager L. (2016). Advances in the management of osteosarcoma. F1000Res..

[29] Kansara M., Teng M., Smyth M., Thomas D. (2014). Translational biology of osteosarcoma. Nat. Rev. Cancer.

[30] Perkins R. (2024). WNT5B drives osteosarcoma stemness, chemoresistance and metastasis. Clin. Transl. Med..

[31] Gomez C. (2009). Hyaluronic acid and HYAL-1 in prostate biopsy specimens: predictors of biochemical recurrence. J. Urol..

[32] Bharadwaj A., Rector K., Simpson M. (2007). Inducible hyaluronan production reveals differential effects on prostate tumor cell growth and tumor angiogenesis. J. Biol. Chem..

[33] Eyster C. (2009). Discovery of new cargo proteins that enter cells through clathrin-independent endocytosis. Traffic..

[34] Siegel R., Miller K., Wagle N., Jemal A. (2023). Cancer statistics, 2023. CA Cancer J. Clin..

[35] Ciardiello F. (2022). Clinical management of metastatic colorectal cancer in the era of precision medicine. CA Cancer J. Clin..

[36] Man Y. (2024). Identification and validation of a novel six-gene signature based on mucinous adenocarcinoma-related gene molecular typing in colorectal cancer. Discov. Oncol..

[37] Jin Z. (2019). The suppressive role of HYAL1 and HYAL2 in the metastasis of colorectal cancer. J. Gastroenterol. Hepatol..

[38] Jiang Z., Li X., Hu L., Jiang Z. (2025). Succinylation-related molecular activities in cancer: metabolic adaptations, immune landscape, and prognostic significance in colorectal cancer. Front. Immunol..

[39] Amane J., Daisuke K., Takashi K., Naoko T., Kohei S. (2024). Current landscape of targeted therapy in esophageal squamous cell carcinoma. Curr. Probl. Cancer.

[40] Zhibin L., Myoung Ok K. (2025). Evolving therapeutic strategies in esophageal squamous cell carcinoma: advances and perspectives. J. Cancer Prev..

[41] Yuan J., Hou B., Guo K., Zhu J., Xiao H. (2024). Tumor-derived exosomal hyaluronidase 1 induced M2 macrophage polarization and promoted esophageal cancer progression. Exp. Cell Res..

[42] Lola R. (2014). Projecting cancer incidence and deaths to 2030: the unexpected burden of thyroid, liver, and pancreas cancers in the United States. Cancer Res..

[43] Divya T., Prakash R. (2019). Tumor-stromal crosstalk in pancreatic cancer and tissue fibrosis. Mol. Cancer.

[44] Agnieszka A R. (2016). Heterogeneous stromal signaling within the tumor microenvironment controls the metastasis of pancreatic cancer. Cancer Res..

[45] Naoki I., Lisheng Z., Koji K. (2008). Impact of the hyaluronan-rich tumor microenvironment on cancer initiation and progression. Cancer Sci..

[46] Takuya O. (2021). Hypoxia increases KIAA1199/CEMIP expression and enhances cell migration in pancreatic cancer. Sci. Rep..

[47] Krishan K. (2023). Targeting BET proteins decreases hyaluronidase-1 in pancreatic cancer. Cells.

[48] V M., A S., HP E., HF K. (1992). Hyaluronan is a secretory product of human pancreatic adenocarcinoma cells. Eur. J. Cell Biol..

[49] Xiao-Bo C. (2020). Negative regulation between the expression levels of receptor for hyaluronic acid-mediated motility and hyaluronan leads to cell migration in pancreatic cancer. Oncol. Lett..

[50] Animesh S. (2014). Demographic and clinical profile of patients with brain metastases: a retrospective study. Asian J. Neurosurg..

[51] P H., M D., D Y., J B. (1996). Differential synthesis and binding of hyaluronan by human breast cancer cell lines. Oncol. Rep..

[52] Isabell W., Leticia O.-F., Klaus P., Volkmar M., Harriet W. (2016). Breast cancer brain metastases: biology and new clinical perspectives. Breast. Cancer Res..

[53] Anastasia G T. (2019). Hyaluronan: molecular size-dependent signaling and biological functions in inflammation and cancer. FEBS. J..

[54] Isabell W. (2017). Role of HYAL1 expression in primary breast cancer in the formation of brain metastases. Breast. Cancer Res. Treat..

[55] Fabienne H. (2022). Key role of hyaluronan metabolism for the development of brain metastases in triple-negative breast cancer. Cells.

[56] Theodoros T K. (2021). Salicylate suppresses the oncogenic hyaluronan network in metastatic breast cancer cells. Matrix. Biol. Plus..

[57] Ina S. (2020). Hyaluronan metabolism is associated with DNA repair genes in breast and colorectal cancer. Screening of potential progression markers using qPCR. Biomedicines..

[58] Fuli W. (2008). HYAL1 and HYAL2 inhibit tumour growth in vivo but not in vitro. PLoS. One.

[59] Li M. (2023). DNA methylation status of HYAL1 in malignant and benign thyroid nodules. Horm. Metab. Res..

[60] Sapudom J. (2020). Biomimetic tissue models reveal the role of hyaluronan in melanoma proliferation and invasion. Biomater. Sci..

[61] Riecks J. (2022). The hyaluronan-related genes HAS2, HYAL1-4, PH20 and HYALP1 are associated with prognosis, cell viability and spheroid formation capacity in ovarian cancer. J. Cancer Res. Clin. Oncol..

[62] Liu Y. (2021). Identification of prognostic biomarkers originating from the tumor stroma of Betel Quid-associated oral cancer tissues. Front. Oncol..

[63] Dong Y. (2021). Urine biomarkers for the diagnosis of bladder cancer: a network meta-analysis. Urol. J..

[64] Roy R., Mandal S., Chakrabarti J., Saha P., Panda C. (2021). Downregulation of hyaluronic acid-CD44 signaling pathway in cervical cancer cell by natural polyphenols Plumbagin, Pongapin and Karanjin. Mol. Cell Biochem..

[65] Thomas R C. (2021). The matrix in cancer. Nat. Rev. Cancer.

[66] Bryan P T. (2004). Hyaluronan: from extracellular glue to pericellular cue. Nat. Rev. Cancer.

[67] Sonia G. (2010). Hyaluronidase expression by an oncolytic adenovirus enhances its intratumoral spread and suppresses tumor growth. Mol. Ther..

[68] Sophia Z S., David M M., Kevin S E., Howard L K. (2023). Therapy with oncolytic viruses: progress and challenges. Nat. Rev. Clin. Oncol..

[69] Shiqun W. (2023). Oncolytic viruses engineered to enforce cholesterol efflux restore tumor-associated macrophage phagocytosis and anti-tumor immunity in glioblastoma. Nat. Commun..

[70] Shibing W., Yuxin L., Chuning X., Jie D., Jiwu W. (2024). An oncolytic vaccinia virus encoding hyaluronidase reshapes the extracellular matrix to enhance cancer chemotherapy and immunotherapy. J. ImmunOther Cancer.

[71] Luo J. (2020). Noncovalently caged firefly luciferins enable amplifiable bioluminescence sensing of hyaluronidase-1 activity in vivo. ACS. Sens..

